# Amplification and generation of ultra-intense twisted laser pulses via stimulated Raman scattering

**DOI:** 10.1038/ncomms10371

**Published:** 2016-01-28

**Authors:** J. Vieira, R. M. G. M. Trines, E. P. Alves, R. A. Fonseca, J. T. Mendonça, R. Bingham, P. Norreys, L. O. Silva

**Affiliations:** 1 GoLP/Instituto de Plasmas e Fusão Nuclear, Instituto Superior Técnico, Universidade de Lisboa, 1049-001 Lisbon, Portugal; 2 Central Laser Facility, STFC Rutherford Appleton Laboratory, Didcot OX11 0QX, UK; 3 DCTI/ISCTE Lisbon University Institute, 1649-026 Lisbon, Portugal; 4 Department of Physics, 107 Rottenrow East, Glasgow G4 0NG, UK; 5 Department of Physics, University of Oxford, Oxford OX1 3PU, UK

## Abstract

Twisted Laguerre–Gaussian lasers, with orbital angular momentum and characterized by doughnut-shaped intensity profiles, provide a transformative set of tools and research directions in a growing range of fields and applications, from super-resolution microcopy and ultra-fast optical communications to quantum computing and astrophysics. The impact of twisted light is widening as recent numerical calculations provided solutions to long-standing challenges in plasma-based acceleration by allowing for high-gradient positron acceleration. The production of ultra-high-intensity twisted laser pulses could then also have a broad influence on relativistic laser–matter interactions. Here we show theoretically and with *ab initio* three-dimensional particle-in-cell simulations that stimulated Raman backscattering can generate and amplify twisted lasers to petawatt intensities in plasmas. This work may open new research directions in nonlinear optics and high–energy-density science, compact plasma-based accelerators and light sources.

## Abstract (web summary)

High intensity light with a non-zero orbital angular momentum could aid the development of laser-wakefield particle accelerators. Here, the authors theoretically show that stimulated Raman backscattering in plasmas can generate and amplify orbital angular momentum lasers to petawatt intensities.

## Graphical abstract

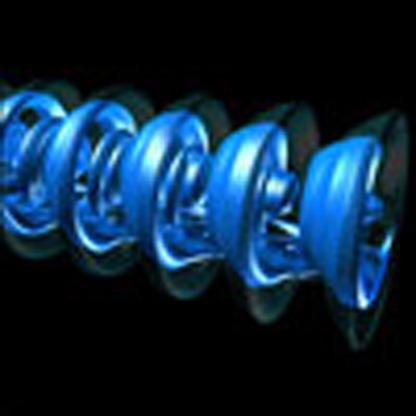

The seminal work by Allen *et al*.^1^ on lasers with orbital angular momentum (OAM) has initiated a path of significant scientific developments that can potentially offer new technologies in a growing range of fields, including microscopy^2^ and imaging^3^, atomic^4^ and nano-particle manipulation^5^, ultra-fast optical communications^6^,^7^, quantum computing^8^ and astrophysics^9^. At intensities beyond material breakdown thresholds, it has been recently shown through theory and simulations that intense (with ≳10^18^ W cm^−2^ intensities) and short (with 10–100 fs durations) twisted laser beams could also excite strongly nonlinear plasma waves suitable for high-gradient positron acceleration in plasma accelerators^10^. As a result of their importance, many techniques have emerged to produce Laguerre–Gaussian lasers over a wide range of frequencies^11^. Common schemes use spiral phase plates or computer-generated holograms to generate visible light with OAM, nonlinear optical media for high-harmonic generation and emission of XUV OAM lasers^12^,^13^ or spiral electron beams in free-electron lasers to produce OAM X-rays^14^,^15^.

Optical elements such as spiral phase plates are designed for the production of laser beams with pre-defined OAM mode contents. Novel and more flexible mechanisms capable of producing and amplifying beams with arbitrary, well-defined OAM states, using a single optical component, would then be interesting from a fundamental point of view, while also benefiting experiments where OAM light is relevant. In addition, the possibility of extending these mechanisms to the production and amplification of laser pulses with relativistic intensities, well above the damage thresholds of optical devices, could also open exciting perspectives for high-energy-density science and applications. The use of a plasma as the optical medium is a potential route towards the production of OAM light with relativistic intensities. Although other routes may be used to produce high-intensity OAM laser pulses, for instance, by placing spiral phase plates either at the start or at the end of a laser amplification chain^16^,^17^, the use of plasmas can potentially lead to the amplification of OAM light to very high powers and intensities. Plasmas also allow for greater flexibility in the level of OAM in the output laser beam than other more conventional techniques.

Here we show that stimulated Raman scattering processes in nonlinear optical media with a Kerr nonlinearity can be used to generate and to amplify OAM light. Plasmas, optical fibres and nonlinear optical crystals are examples of nonlinear optical media with Kerr nonlinearity. Although optical parametric oscillators have also been used to transfer OAM from a pump to down converted beams^18^, here we explore the creation of new OAM states absent from the initial configuration, according to simple selection rules. We also demonstrate that stimulated Raman scattering processes can generate and amplify OAM light even in scenarios where no net OAM is initially present. To this end, we use an analytical theory, valid for arbitrary transverse laser field envelope profiles, complemented by the first three-dimensional (3D) *ab initio* particle-in-cell (PIC) simulation of the process using the PIC code OSIRIS^19^, considering that the optical medium is a plasma. Starting from recent experimental and theoretical advances^20^,^21^,^22^, our simulations and theoretical developments show that stimulated Raman processes could pave the way to generate OAM light in nonlinear optical media and that the nonlinear optics of plasmas^23^,^24^ could provide a path to generate and amplify OAM light to relativistic intensities^25^,^26^,^27^,^28^.

## Results

### Theoretical model

We illustrate our findings considering that the nonlinear optical medium is a plasma. Extension to other materials is straightforward. In a plasma, stimulated Raman backscattering is a three-wave mode coupling mechanism in which a pump pulse (frequency *ω*_0_ and wavenumber *k*_0_), decays into an electrostatic, or Langmuir, plasma wave (frequency *ω*_p_ and wavenumber 2*k*_0_−*ω*_p_/*c*) and into a counter-propagating seed laser (frequency *ω*_1_=*ω*_0_−*ω*_p_ and wavenumber *k*_1_=*ω*_p_/*c*−*k*_0_). The presence of OAM in the pump and/or seed results in additional matching conditions that ensure the conservation of the angular momentum carried by the pump when the pump itself decays into a scattered electromagnetic wave and a Langmuir wave^29^. These additional matching conditions, which are explored in more detail in Supplementary Notes 1–4 and Supplementary Figs 1–3), correspond to selection rules for the angular momentum carried by each laser and plasma wave. Here we illustrate key properties of OAM generation and amplification by exploring different seed and pump configurations.

In order to derive a model capable of predicting stimulated Raman scattering OAM selection rules, we start with the general equations describing stimulated Raman scattering, given by 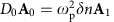, 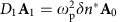 and 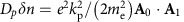, where 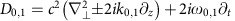, *D*_*p*_=2*iω*_p_∂_*t*_, and where the minus (−) sign is used to describe the seed pulse evolution. Moreover, **A**_0,1_ is the envelope of the pump/seed laser, with complex amplitude, given by 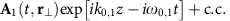, where *t* is the time and *z* the propagation distance. We note that **A**_0,1_ are arbitrary functions of the transverse coordinate **r**_⊥_. The complex amplitude of the plasma density perturbations is 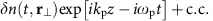, where *k*_p_=*ω*_p_/*c* is the plasma wavenumber, 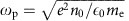 the plasma frequency, *m*_e_ the mass of the electron, 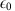 the vacuum electric permittivity and *e* the elementary charge. Although these general equations can be used to retrieve the selection rules for the OAM that will be explored throughout this paper, it is possible to derive exact solutions in the long pulse limit, where 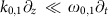 and in the limit where the pump laser contains much more energy than the seed laser energy. In this case, since 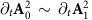, and 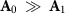 (pump has more energy than seed), then 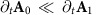 (this condition is strictly satisfied in our simulations when new modes are created and until their energy becomes comparable to the energy in the pump pulse). In this case, it is possible to show that stimulated Raman scattering of a seed beam **A**_1_ from a pump beam **A**_0_ creates a plasma wave density perturbation, given by:


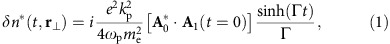



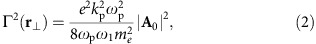


where Γ is the growth rate at which the plasma amplitude grows as the interaction progresses and **r**_⊥_ is the transverse position. The amplification of the seed is given by:


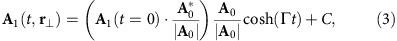


where (*ω*_1_, *k*_1_) are the frequency and wavenumber of the seed laser pulse, respectively, and *C* is a constant of integration. The derivation of equations (1)–(3), 14, 15, presented in detail in Supplementary Note 1, assumes that the pump and the seed satisfy the frequency matching conditions stated above, being valid for arbitrary transverse laser envelope profiles as long as the paraxial equation is satisfied. Neglecting pump depletion does not change the selection rules for the OAM, as discussed in the remainder of this work. Unless explicitly stated, the generic expression for the pump vector potential (or electric field) is 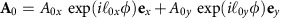, where (**e**_*x*_, **e**_*y*_) are the unit vectors in the transverse *x* and *y* directions, and *φ* the azimutal angle. Similarly, the generic expression for the seed vector potential is 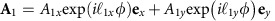. Selection rules can then be generally derived by inserting these expressions into the factor (**A**_1_(*t*=0) ˙ **A**_0_*)**A**_0_ in equation (3). Although we have assumed that the plasma is the optical medium, other nonlinear optical media with Kerr nonlinearity will also exhibit similar phenomena.

### PIC simulations

**Figure 1:**
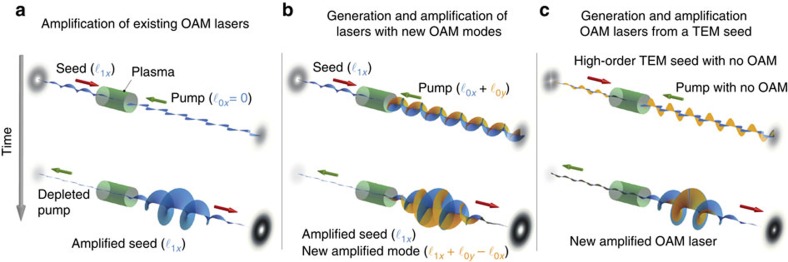
Generation and amplification of OAM lasers via stimulated Raman backscattering. In Raman amplification, a long pump laser transfers its energy to a short, counter-propagating seed laser in a plasma. The process depletes the pump laser pulse energy and enhances the intensity of the seed laser. The seed/pump lasers propagate in the direction of the red/green arrow, respectively. Polarization in the *x*/*y* direction is represented by blue/orange lasers, respectively. The position of the plasma, relative to the seed and pump lasers, is shown by the green cylinders. The back/front projections show the intensity profile of the closest laser. (**a**) A set-up leading to the amplification of a seed with OAM. (**b**) The generation and amplification of new OAM modes. (**c**) The generation and amplification of a new OAM laser in a configuration with no initial OAM.

We will now use equation (3) to explore OAM generation and amplification in three separate classes of initial set-ups, all identified in Fig. 1. We start by studying the case of the amplification of existing OAM modes. Figure 1a illustrates the process in a set-up leading to the amplification of a seed in an arbitrary, single state of OAM 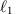 in a plasma using a counter-propagating Gaussian pump laser without OAM. The mechanism is trivially generalized for a pump with arbitrary OAM 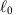. We can then assume a pump linearly polarized in the *x* direction with OAM 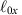, which decays into a Langmuir plasma wave with OAM 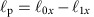 and into a seed, also linearly polarized in the *x* direction with OAM 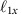. Making these substitutions into equation (3) confirms that the amplification of *A*_1_ retains the initial seed OAM. For the specific example in Fig. 1a, where 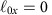, direct substitution of 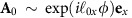 and 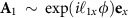 in equation (1) shows that the plasma wave density perturbations 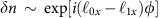 have OAM 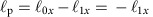, that is, the plasma wave absorbs the excess OAM that may exist between the pump and the seed (see Supplementary Note 2 and Supplementary Fig. 1 for several examples illustrating angular and linear momentum matching conditions, demonstrating that the plasma wave always absorbs the excess OAM between pump and seed.) The scheme is thus ideally suited to amplify an existing OAM seed using a long Gaussian pump without OAM. The amplification of circularly polarized OAM lasers, with both spin and OAM, obeys similar selection rules. For amplification to occur in this case, and similar to stimulated Raman backscattering of circularly polarized Gaussian lasers, both seed and pump need to be polarized with the same handedness either in **e**_+_=**e**_*x*_+*i***e**_*y*_ or in **e**_−_=**e**_*x*_−*i***e**_*y*_.

**Figure 2:**
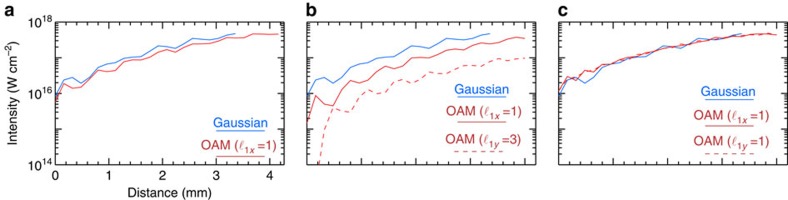
Simulation results showing the generation and amplification of OAM lasers. Blue refers to the amplification of a Gaussian seed by a Gaussian pump. The initial laser configuration in each panel (**a**–**c**) corresponds to the initial set-up illustrated by each corresponding panel (**a**–**c**) in Fig. 1. (**a**) The amplification of a seed with 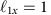 using a long Gaussian pump (red). (**b**) The generation and amplification of a new OAM mode with 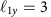 (dashed red) and of an existing mode with 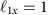 (solid red) from an OAM pump polarized in two directions with 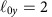 and 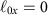. (**c**) The amplification of a new OAM mode with 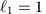 from a TEM seed with no net initial OAM and from a Gaussian pump. See Table 1 for simulation parameters.

Figure 2a illustrates 3D simulation results showing the amplification of an 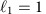, linearly polarized seed from a linearly polarized Gaussian pump. Simulation parameters are stated in Table 1. Figure 2a shows that the growth rate for the amplification process is nearly indistinguishable from stimulated Raman amplification of Gaussian lasers. In agreement with equation (2), this result also indicates that, in general, the overall amplification process is OAM-independent.

Stimulated Raman scattering also provides a mechanism to create new OAM modes (that is, modes that are absent from the initial pump/seed lasers) and amplify them to very high intensities. Figure 1b illustrates the process schematically. The pump electric fields can have different OAM components in both transverse directions *x* and *y*. Each component is represented in blue and orange in Fig. 1b. The pump electric field component in *x* has OAM 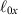. The pump electric field component in *y* has OAM 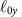. The initial seed electric field contains an OAM 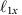 component in the *x* direction. After interacting in the plasma, the pump becomes depleted and a new electric field component appears in the seed with OAM, given by 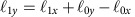.

The process can be physically understood by examining the couplings between the plasma and light waves in the example considered above. Initially, a plasma wave will be excited due to beating pump and seed components that have their electric fields pointing in the transverse *x* direction. According to equation (1), the plasma wave OAM is 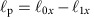. This plasma wave ensures OAM conservation for the pump and seed electric field components in the *x* direction. The (same) plasma wave also couples the pump and seed modes with electric field components pointing in the *y* direction. Thus, 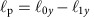 must also hold in order to ensure conservation of angular momentum. This implies the generation of a new seed component with electric field polarized in *y* so that OAM is conserved at all times and in both components. The OAM of the new seed component is thus 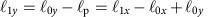.

Alternatively, this selection rule can also be found by examining equation (3). Direct substitution of a pump profile with 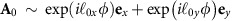 and of an initial seed profile with 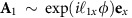 then leads to the generation of a new seed with 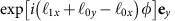. The same selection rules would also hold if the pump consists of combination of a right- and left-handed circularly polarized modes, each with different OAM, and the seed initially contains only a left- or right-handed circularly polarized mode. In this case, a new seed component would appear with right- or left-handed circular polarization. The new mode is created to ensure conservation of OAM. The selection rules are identical as long as polarizations in **e**_*x*_/**e**_*y*_ are replaced by polarizations in **e**_+_/**e**_−_ (where **e**_±_=**e**_*x*_±*i***e**_*y*_). This set-up provides a robust mechanism for the production and amplification of a new and well-defined OAM mode, absent from the initial set of lasers. The generation of a new OAM mode when a linearly polarized seed interacts with a pump with electric field components in the two orthogonal directions is also illustrated in Supplementary Note 3 and Supplementary Fig. 2.

**Figure 3:**
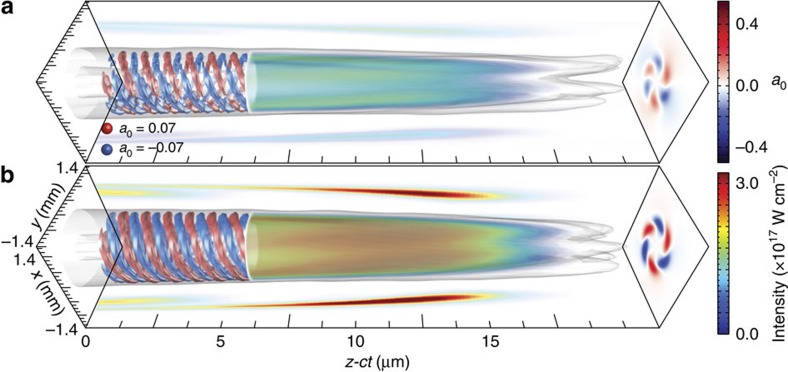
Simulation results showing the generation and amplification of a new OAM modes. The new mode with 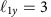 grow from a seed with 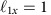 and a linearly polarized pump with a Gaussian profile in the *x* direction, and with an OAM 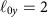 in the *y* direction. *z*=2 mm (**a**) and *z*=6.22 mm (**b**). The initial set-up is illustrated in Fig. 1b. Projections in the (*x*,*z-ct*) and (*y*,*z-ct*) planes show intensity profile slices at the mid-plane of the OAM mode (blue–green–red colours). Projections in the (*x*,*y*) plane (blue–white–red) show the normalized vector potential (*a*_0_) field envelope of the new OAM mode at the longitudinal slice where the laser intensity is maximum. The envelope of the 3D laser intensity is also shown for *z*>6.25 mm in blue–green–red colours, and normalized vector potential isosurfaces for *z*<6.25 mm in blue and red.

Figure 3 shows a result of a 3D PIC simulation illustrating the production of a new seed mode with 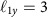, which is initially absent from the simulation, from a pump with 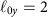, 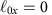 and an initial seed with 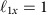 (simulation parameters given in Table 1). Figure 3 presents several distinct signatures of the new OAM mode with 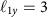. The laser vector potential shows helical structures, which indicate that the new mode has OAM. The normalized vector potential forms a pattern that repeats each 3 turns, which turn in the clockwise direction from the front to the back of the pulse, a signature for 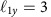. Field projections in the (x,y) plane also show a similar pattern further confirming that the new OAM mode has 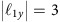. The change in colour from blue–green in (a) to (green–red) in (b) is a clear signature for the intensity amplification of the new seed mode. Intensity was calculated using 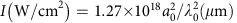, where *λ*_0_=0.8 μm is the central laser wavelength. Figure 3 also shows that the amplified laser envelope acquires a bow-shaped profile^21^,^22^,^30^, a key signature of Raman amplification identified in ref. 21. In agreement with theory (equations (2) and (3)), Fig. 2b shows that the new 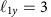 mode and the existing 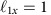 OAM mode amplify at nearly coincident growth rates. Still, since it grows from initially higher intensities, the existing 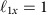 mode reaches higher final intensities than the new OAM mode with 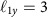. The generation of the new modes in Fig. 2b also illustrates the transition from the regime where the depletion of the pump is negligible (small signal and exponential growth) to the regime where the depletion of the pump is not negligible (strong signal and linear growth). Hence, Fig. 2b shows an exponential growth of the new mode up to *z*<0.6 mm. For *z*>0.6 mm, the energy in the new seed becomes comparable to the energy contained in the pump pulse. As a result, the growth slows down significantly, becoming linear with the propagation distance^26^.

L. Allen *et al*.^1^ showed that particular superpositions of Hermite–Gaussian modes (also called transverse electro-magnetic or TEM modes) are mathematically equivalent to Laguerre–Gaussian modes. Since the transverse amplitude distribution of high-order (transverse) laser modes is usually described by a product of Hermite–Gaussian polynomials, which is also usually associated with TEM modes, this result paved the way for experimental realization of vortex light beams with OAM from existing TEM laser modes. It is thus interesting and important to explore whether and how stimulated Raman backscattering can be used to generate and amplify light with OAM from Hermite–Gaussian laser beams, that is, from initial configurations with no net OAM. Figure 1c illustrates the process. From now on, we refer to each Hermite–Gaussian beam as a TEM_*m*,*n*_ laser, where (*m*, *n*) represents the Hermite–Gaussian mode. The TEM mode electric field is given by equation (5) (see Methods section). We consider first a Gaussian pump linearly polarized at 45°, that is, having similar electric field amplitudes in both transverse directions *x* and *y*. The Gaussian pump can then also be written as **A**_0_∼TEM_00_(**e**_*x*_+**e**_*y*_). In addition, we assume a seed with a TEM_10_ mode electric field component in *x* and with a TEM_01_ mode electric field component in *y*. The two seed modes are *π*/2 out of phase with respect to each other. The seed is given by **A**_1_∼TEM_10_**e**_*x*_+*i*TEM_01_**e**_*y*_. This set-up is represented in Fig. 1c, where blue and orange colours refer to the pump and seed components polarized in the *x* and *y* directions, respectively.

Although this set-up has no initial OAM, since both pump and seed have no OAM, it results in the generation and amplification of an OAM mode with 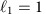. In order to understand the OAM generation mechanism, we first consider equation (1). According to equation (1), the beating between the TEM_10_ seed with the Gaussian pump in the *x* direction will drive a TEM_10_ daughter plasma wave component. The beating between the *i*TEM_01_ seed and Gaussian pump in the *y* direction will drive a *i*TEM_01_ daughter plasma wave component. These two plasma wave components are *π*/2 out of phase with respect to each other. Hence, the resulting plasma wave will be a combination of TEM modes given by *δn*∼TEM_10_−*i*TEM_01_, where the *i* denotes the phase difference between modes. According to ref. 1, this mode combination is equivalent to a Laguerre–Gaussian mode with 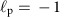. In order to conserve angular momentum, a new seed component with 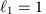 will then have to be generated and amplified in the direction of polarization of the pump. This process is also illustrated in Supplementary Note 4 and Supplementary Fig. 3.

It is also possible to reach this conclusion by substituting in equation (3) the expressions for initial Gaussian pump transverse profile [**A**_0_∼TEM_00_(**e**_*x*_+**e**_*y*_)] and initial TEM seed profile (**A**_1_∼TEM_10_**e**_*x*_+*i*TEM_01_**e**_*y*_). This substitution yields a new seed transverse profile given by **A**_1_∼(TEM_10_+*i*TEM_01_)(**e**_*x*_+**e**_*y*_), corresponding to a Laguerre–Gaussian mode with 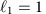 (ref. 1). Similarly, a circularly polarized Gaussian pump and a **A**_1_∼TEM_10_**e**_*x*_+TEM_01_**e**_*y*_ seed (that is, without phase difference between the TEM_10_ and TEM_01_ modes) would also lead to a new seed component with 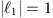. The plasma can then be viewed as a high-intensity mode converter.

**Figure 4:**
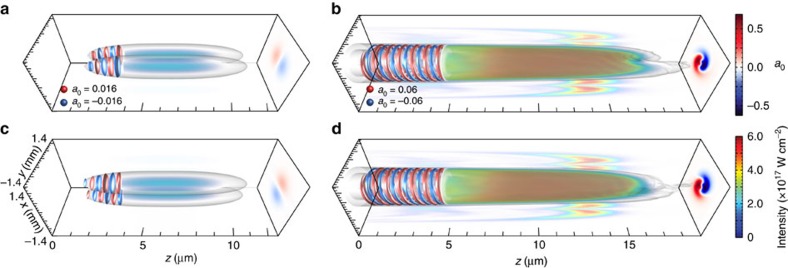
Simulation result showing the generation and amplification of a new OAM mode from initial configurations with no net OAM. The new mode is linearly polarized in x and in y with 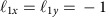 from an initial seed polarized in the *x* direction with a TEM_01_ mode and in the *y* direction with a TEM_10_ mode that is *π*/2 out of phase with respect to the TEM_01_ mode polarized in *x*. The pump is a Gaussian laser linearly polarized at 45°. The initial laser set-up corresponds to Fig. 1c. The meaning of the colour scales and physical quantities plotted in all panels are identical to Fig. 3. The values of the laser vector potential illustrated by the isosurfaces of **a**,**c** are shown by the spheres in **a**. Those iso-surface values for **b**,**d** are indicated in **b**. (**a**,**c**) The initial seed TEM modes in *x*- and *y* directions, respectively. (**b**,**d**) The new OAM mode electric field components at *z*=3.5 mm in *x* (**b**) and *y* (**d**).

Figure 4 shows results from a 3D simulation that confirms these predictions (see Table 1 for simulation parameters). The simulation set-up follows the example of Fig. 1c described earlier. Simulations show that stimulated Raman scattering leads to a new OAM mode with 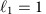 linearly polarized at 45°. Figure 2c shows that the amplification rates are comparable to the other typical scenarios shown in Fig. 2a,b. The change on the field topology of the seed normalized vector potential shown in Fig. 4, from plane isosurfaces to helical isosurfaces, indicates the generation of a laser with OAM from a configuration with no net OAM. Normalized vector potential isosurfaces, and projection in the *yz* direction, form a pattern that repeats each turn and that rotates clockwise from the front to the back of the pulse, thereby indicating an OAM with 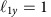.

## Discussion

We have so far assumed that the lasers are perfectly aligned. In experiments, however, the beams can only be aligned within a certain precision. In the presence of misalignments, our results will still hold as long as Raman side-scattering can be neglected, that is, when the angle between the two pulses is much smaller than 90°. The *k*-matching conditions are then still satisfied in the presence of small misalignments because the nonlinear medium, a plasma in the case of our simulations, absorbs any additional transverse wave vector component. Thus, momentum is still locally conserved, thereby allowing for Raman backscatter processes (interestingly, we note that when using OAM beams, the wave vectors of seed and pump are already locally misaligned). Despite lowering the total interaction time, and possibly the final amplification level, a small angle between the seed and pump will not change the OAM selection rules and the overall physics of stimulated Raman scattering.

We note that our seed laser pulse final intensity, on the order of 10^17^ W cm^−2^, and seed laser spot size, on the order of 1 mm, indicate the production and amplification of Petawatt class twisted lasers with OAM. Additional simulations (not shown) revealed the generation and amplification of circularly polarized OAM modes using a scheme similar to that in Fig. 1b. Moreover, simulations showed that Raman amplification can also operate in the absence of exact frequency/wavenumber matching between seed and pump as long as the seed is short so that its Fourier components can still satisfy *k-* and *ω*-matching conditions.

Finally, we note that our results could be extended to other nonlinear optical media with Kerr nonlinearities. In a plasma, the coupling between seed and pump is through an electron Langmuir wave, which also ensures frequency, wavenumber and OAM matching conditions will hold. In other nonlinear optical media, molecular vibrations, for instance, would play the role of the plasma Langmuir wave. We note that the possibility of OAM transfer has been explored in solids^18^. Similar phenomenology as illustrated in this work could also be obtained in three-wave mixing processes, where an idler wave could play the role of the plasma Langmuir wave. One advantage of testing these set-ups in nonlinear Kerr optical media such as a crystal is that lasers with much lower intensities could be used (see Supplementary Note 5 for a discussion in nonlinear optical media with Kerr nonlinearity admitting three-wave interaction processes). The plasma, however, offers the possibility to amplify these lasers to very high intensities. This scheme could also be used in combination with optical pulse chirped pulse amplification to pre-generate and pre-amplify new OAM modes via stimulated Raman scattering before they enter the plasma to be further amplified. Similar configurations (for example, stimulated Brillouin backscattering^31^) can also be envisaged to produce intense OAM light.

## Methods

### Set-up of numerical simulations and simulation parameters

Simulations have been performed using the massively parallel, fully relativistic, electro-magnetic PIC code OSIRIS^19^. In the PIC algorithm, spatial dimensions are discretized by a numerical grid. Electric and magnetic fields are defined in each grid cell and advanced through a finite difference solver for the full set of Maxwell's equations. Each cell contains macro-particles representing an ensemble of real charged particles. Macro-particles are advanced according to the Lorentz force. Since background plasma ion motion is negligible for our conditions, ions have been treated as a positively charged immobile background. The plasma was initialized at the front of the simulation box that moves at the speed of light *c*. Note that although the simulation are performed in a frame that moves at *c*, the moving window corresponds to a Galilean transformation of coordinates where all computations are still performed in the laboratory frame. The simulation box dimensions were 50 × 2,870 × 2,870 μm, it has been divided into 650 × 2,400 × 2,400 cells and each cell contains 1 × 1 × 1 particles (3.7 × 10^9^ simulation particles in total). Additional simulations with 1 × 2 × 2 particles per cell showed no influence on our conclusions and simulation results. The pump laser was injected backwards from the leading edge of the moving window^32^,^33^. In order to conserve canonical momentum, the momentum of each plasma electron macro-particle has been set to match the normalized laser vector potential. The particles are initialized with no thermal spread.

The initial OAM seed and pump laser electric field is given by:


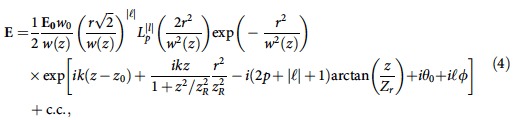


where c.c. denotes complex conjugate, **E**_**0**_=(*E*_0*x*_, *E*_0*y*_) is the laser electric field at the focus, with (*E*_0*x*_, *E*_0*y*_) being the electric field amplitudes in the transverse *x* and *y* directions, respectively. For a linearly polarized laser, there is no phase difference between *E*_0*x*_ and *E*_0*y*_. For circularly polarized light, both components are *π*/2 out of phase, that is, *E*_0*x*_=±*iE*_0*y*_. In addition 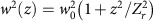 is the waist of the beam as a function of the propagation distance *z* in vacuum, *w*_0_ the waist at the focal plane, 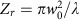 the Rayleigh length, and *λ*=2*πc*/*ω*=2*π*/*k* the central wavelength of the laser, *ω* and *k* are its central frequency and wavenumber, respectively. In addition, 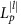 is a generalized Laguerre polynomial with order 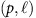, with 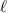 being the index that gives rise to the OAM, 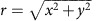 the radial distance to the axis, *θ*_0_ an initial phase and *z*_0_ the centre of the laser. We note that all simulations involving Laguerre–Gaussian modes have *p*=0. The initial electric field of an Hermite–Gaussian (TEM) laser is given by:


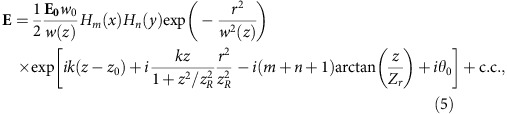


where *H*_*m*_ is an Hermite polynomial of order *m*. Moreover, the wavenumber of the pump laser (which travels in the plasma) in all simulations presented in Figs 2, 3, 4 is set according to the linear plasma dispersion relation 
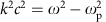
, where 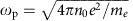 is the plasma frequency associated with a background plasma density *n*_0_, and where *e* and *m*_e_ are, respectively, the elementary charge and electron mass. The seed frequency and wavenumber are set according to the matching conditions for Raman amplification (Table 1).

## Additional information

**How to cite this article:** Vieira, J. *et al*. Amplification and generation of ultra-intense twisted laser pulses via stimulated Raman scattering. *Nat. Commun.* 7:10371 doi: 10.1038/ncomms10371 (2016).

## Supplementary Material

Supplementary Information Supplementary Figures 1-3, Supplementary Notes 1-5 and Supplementary References.

## Figures and Tables

**Table 1:** Laser parameters for the different Raman Amplification regimes to generate and amplify OAM lasers.

	**Amplification of existing modes**		**Generation and amplification of new OAM modes**			
	**Pump**	**Seed**	**OAM seed**		**TEM seed**	
			**Pump**	**Seed**	**Pump**	**Seed**
TEM	—	—	—	—	TEM_00_**e**_*x*_+TEM_00_**e**_*y*_	TEM_01_**e**_*x*_+iTEM_10_**e**_*y*_
OAM	L_00_**e**_*x*_	L_01_**e**_*x*_	L_00_**e**_*x*_+L_02_**e**_*y*_	L_01_**e**_*x*_	—	—
*a*_0_ (peak)	0.02	0.06	0.02	0.03	0.02	0.08
Spot (μm)	718	435	718	435	718	435
Duration (fs)	25 × 10^3^	25	25 × 10^3^	25	25 × 10^3^	25
*w*_0_/*w*_p_	20	19	20	19	20	19

Simulation parameters are close to ideal Raman amplification regimes determined in ref. 21. In all simulations, the probe has a central wavelength of 1 μm. The background plasma density is *n*_0_=4.3 × 10^18^ cm^−3^ for all simulations presented. When the pump/probe initially has components in both the transverse directions, the initial peak *a*_0_, spot size and durations present in the table are identical for every component. 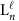 refers to a Laguerre–Gaussian 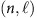 mode, where the OAM corresponds to the index 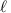. TEM_*mn*_ correspond to Hermite–Gaussian lasers with order (*m*, *n*). The table only describes the initial simulation conditions.
