# Supplementary material for: Amplification and generation of ultra-intense twisted laser pulses via stimulated Raman scattering
Source: Nat Commun. 2016 Jan 28;7:10371. doi: 10.1038/ncomms10371 (PMC4738314; doi:10.1038/ncomms10371)
Supplement: Supplementary Information — Supplementary Figures 1-3, Supplementary Notes 1-5 and Supplementary References. [file ncomms10371-s1.pdf]

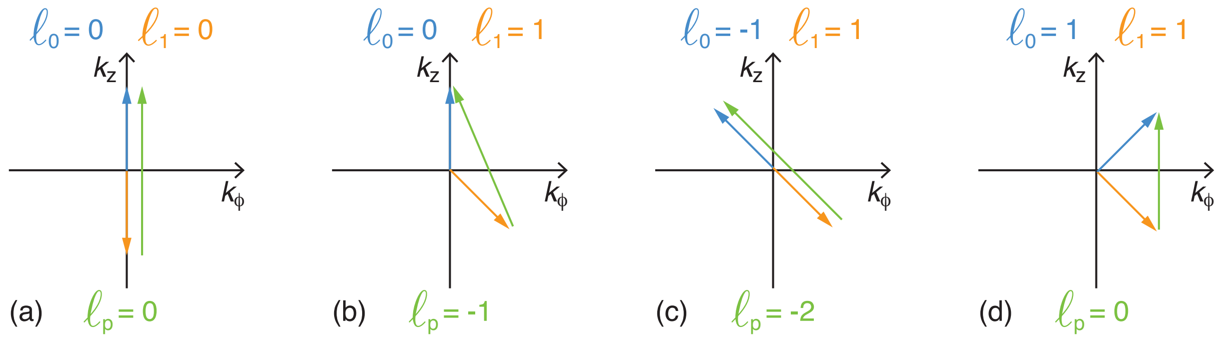

Supplementary Figure 1. Illustration of the angular momentum selection rules for stimulated Raman backscattering amplification for various configurations. The vertical axis refers to the wave number in the propagation direction, and the horizontal azis to the azimuthal wavenumber corresponding to the orbital angular momentum. Blue refers to the pump, orange to the seed and green to the daughter plasma (Langmuir) wave. In addition, subscripts 0, 1 and p in every quantity correspond to the pump, seed and plasma wave. (a) shows Raman amplification matching conditions without OAM. (b) Raman amplification for of an OAM seed by a Gaussian pump. (c)-(d) Raman amplification for a scenario where both seed and pump have anti-parallel OAM, and parallel angular momenta, respectively.

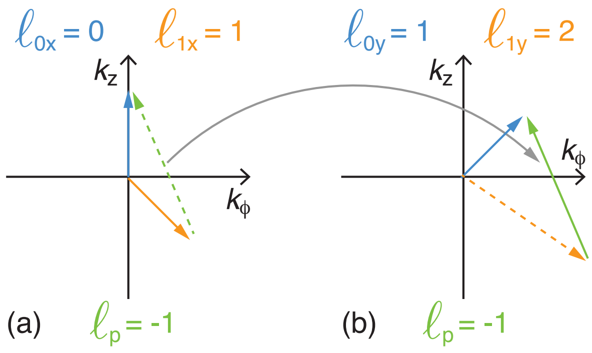

Supplementary Figure 2. Illustration of the generation and amplification of a new OAM mode using stimulated Raman backscattering. The colours have the same meaning as in Fig. 1. Dashed lines indicate the new modes that are created at each step. The grey arrows indicate the coupling of plasma waves to the lasers polarised both in the x and y directions. The figure illustrates an initial configuration where the pump is polarised in x with a mode with OAM given by $\ell_{0x} = 0$ and $\ell_{0y} = 1$. In addition, the initial seed is polarised in y with $\ell_{1x} = 1$. Panels (a) and (b) show the wavenumber matching conditions for the x and y directions respectively.

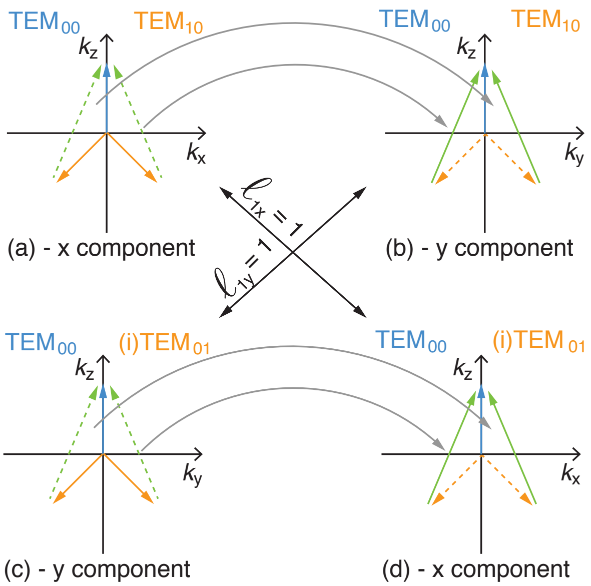

Supplementary Figure 3. Generation of OAM light from an initial configuration with no OAM. The initial pump is a Gaussian in x and y, and the initial seed is a TEM_{10} in x and TEM_{01} in y. The (i) in (a) and (d) denote TEM seed components that are $\pi/2$ out of phase. Dashed lines indicate the new modes that are created at each step. The grey arrows indicate the coupling of plasma waves to the lasers polarised both in the x and y directions. The black arrows in diagonal indicate the coupling between the different laser modes that result in new OAM modes. Panels (a) and (c) show the wavenumber matching conditions in x and in y for the pump and seed beams that are initially present. Panels (b) and (d) show the wavenumber matching conditions in the y and x directions for the new modes that are created.

SUPPLEMENTARY NOTE 1 - STIMULATED RAMAN BACKSCATTERING

To derive Orbital Angular Momentum (OAM) selection rules for Raman amplification we write pump and seed laser vector potentials as $\mathbf{A}_{\mathrm{pump}} = \mathbf{A}_0(r,\phi,t)\exp(ik_0z - i\omega_0t) + c.c.$ and $\mathbf{A}_{\mathrm{seed}} = \mathbf{A}_1(r,\phi,t)\exp(-ik_1z - i\omega_1t) + c.c.$, where $\mathbf{A}_0(\mathbf{r})$ and $\mathbf{A}_1(\mathbf{r})$ are arbitrary functions of the transverse coordinate $\mathbf{r}$, being slowly varying envelopes for the pump ($\mathbf{A}_0$) and seed ($\mathbf{A}_1$) respectively, and where (k_0,ω_0) and (k_1,ω_1) are the pump and seed lasers wavenumber (k) and frequency (ω) respectively. Note that we use k_0 and $-k_1$ to indicate that the pulses travel in opposite directions. In addition, we write the plasma electron density perturbation (Langmuir wave) as $(n_e - n_0)/n_0 = \delta n \exp(ik_{\mathrm{p}}z - i\omega_{\mathrm{p}}t) + c.c.$, where δn is a slowly varying

envelope, and where $(k_\mathrm{p}, \omega_\mathrm{p})$ are, respectively, the plasma wavenumber ($k_\mathrm{p} \approx 2k_0 - \omega_\mathrm{p}/c$) and frequency $\omega_\mathrm{p} = \sqrt{e^2 n_0/\epsilon_0 m_e}$, with c the speed of light, e the elementary charge, n_0 the background plasma density, ϵ_0 the vacuum electric permittivity, and m_e the electron mass. We use cylindrical coordinates where (r, ϕ, z) are the radial distance to the axis (r), the azimuthal angle (ϕ), and the longitudinal distance (z). Using the slowly varying envelope approximation, the following equations describing Raman backscattering can be derived [1–4]:

$$D_0 \mathbf{A}_0 = \omega_\mathrm{p}^2 \delta n \mathbf{A}_1 \tag{1}$$

$$D_1 \mathbf{A}_1 = -\omega_\mathrm{p}^2 \delta n^* \mathbf{A}_0 \tag{2}$$

$$D_\mathrm{p} \delta n = \frac{e^2 k_\mathrm{p}^2}{2m_e^2} \left(\mathbf{A}_0 \cdot \mathbf{A}_1^*\right), \tag{3}$$

where the superscript * denotes the complex conjugate and where the operators D_0, D_1 and D_p are given by:

$$D_0 = c^2 \left(\nabla_\perp^2 + 2ik_0 \frac{\partial}{\partial z}\right) + 2i\omega_0 \frac{\partial}{\partial t} \tag{4}$$

$$D_1 = c^2 \left(\nabla_\perp^2 - 2ik_1 \frac{\partial}{\partial z}\right) + 2i\omega_1 \frac{\partial}{\partial t} \tag{5}$$

$$D_\mathrm{p} = 2i\omega_\mathrm{p} \frac{\partial}{\partial t}, \tag{6}$$

where Eq. (6) is strictly valid for cold plasmas. In addition to Eqs. (1-6), the lasers and plasma wavenumber and frequency matching conditions are given by $k_0 = k_1 + k_\mathrm{p}$, $\omega_0 = \omega_1 + \omega_\mathrm{p}$. Each laser also obeys the dispersion relation of electromagnetic waves in a plasma, where $k_{0,1}^2 c^2 = \omega_{0,1}^2 - \omega_\mathrm{p}^2$.

We can simplify Eq. (1-3) by making a general assumption that the vector potential envelope of each laser can be written as $\mathbf{A} = A_\mathrm{x}(t,z) T_\mathrm{x}(\mathbf{r}_\perp, z)\mathbf{e}_\mathrm{x} + A_\mathrm{y}(t,z) T_\mathrm{y}(\mathbf{r}_\perp, z)\mathbf{e}_\mathrm{y}$, where $A_\mathrm{x/y}$ is a function of t and z and represents the longitudinal envelope profile, and where $T_\mathrm{x/y}$ is a function of the coordinates $\mathbf{r}_\perp$ and z, and represents the transverse envelope profile. We then use $A_{0,\mathrm{x/y}} T_{0,\mathrm{x/y}}$ and $A_{1,\mathrm{x/y}} T_{1,\mathrm{x/y}}$ to designate the pump and seed fields in the transverse x and y directions respectively. We can also assume that plasma density perturbations can be written as $\delta n = \delta\hat{n}(t,z) T_{\delta n}$. Although our calculations are valid for any $T_\mathrm{x,y}$, for a Laguerre-Gaussian mode with OAM level ℓ and radial mode p, $T_\mathrm{x,y}$ (or $T_{\delta n}$) is given by:

$$T = \mathrm{LG}_p^{|\ell|}(\mathbf{r}_\perp, z) \exp\left[\frac{ikz}{(1 + z^2/z_r^2)} \frac{\mathbf{r}_\perp^2}{z_r^2} + i\ell\phi\right] \exp[-i\zeta(z)] + c.c., \tag{7}$$

where we dropped the subscripts for simplicity, where

$$\mathrm{LG}_p^{|\ell|}(\mathbf{r}_\perp, z) = \frac{w_0}{2w(z)}\left(\frac{r\sqrt{2}}{w(z)}\right)^{|\ell|} L_p^{|\ell|}\left(-\frac{2r^2}{w^2(z)}\right)\exp\left(-\frac{r^2}{w^2(z)}\right), \tag{8}$$

is the Laguerre-Gaussian polynomial with azimutal index ℓ and radial index p, and where

$$\zeta(z) = (2p + |\ell| + 1)\arctan(z/z_r), \tag{9}$$

is the Gouy phase shift. In addition, $z_r = kw_0^2/2$ is the Rayleigh length, k is the wavenumber, w_0 is the spot-size at focus, and $w(z) = w_0(1 + z^2/z_r^2)^{1/2}$.

Since $\nabla_\perp A_\mathrm{x} = \nabla_\perp A_\mathrm{y} = 0$ as A_x and A_y are functions of (t, z) only, and since $\partial T_{\mathrm{x/y}}/\partial t = 0$ because $T_{\mathrm{x/y}}$ depends on $(\mathbf{x}_\perp, z)$ only, inserting Eq. (7) into Eq. (4) and (5) yields:

$$D_0 A_{0,\mathrm{x/y}} = c^2\left(A_{0,\mathrm{x/y}}\nabla_\perp^2 T_{0,\mathrm{x/y}} + 2ik_0 A_{0,\mathrm{x/y}}\frac{\partial T_{0,\mathrm{x/y}}}{\partial z} + 2ik_0 T_{0,\mathrm{x/y}}\frac{\partial A_{0,\mathrm{x/y}}}{\partial z}\right) + 2i\omega_0 T_{0,\mathrm{x/y}}\frac{\partial A_{0,\mathrm{x/y}}}{\partial t}, \tag{10}$$

$$D_1 A_{1,\mathrm{x/y}} = c^2\left(A_{1,\mathrm{x/y}}\nabla_\perp^2 T_{1,\mathrm{x/y}} - 2ik_1 A_{1,\mathrm{x/y}}\frac{\partial T_{1,\mathrm{x/y}}}{\partial z} - 2ik_1 T_{1,\mathrm{x/y}}\frac{\partial A_{1,\mathrm{x/y}}}{\partial z}\right) - 2i\omega_1 T_{1,\mathrm{x/y}}\frac{\partial A_{1,\mathrm{x/y}}}{\partial t}. \tag{11}$$

To explore all selection rules we can assume that the transverse laser envelopes T_0 and T_1 obey to the paraxial approximation:

$$c^2\left(\nabla_\perp^2 + 2ik_0\frac{\partial}{\partial z}\right)T_{0,\mathrm{x/y}} \approx 0, \tag{12}$$

$$c^2\left(\nabla_\perp^2 - 2ik_1\frac{\partial}{\partial z}\right)T_{1,\mathrm{x/y}} \approx 0. \tag{13}$$

Using Eqs.(12) and (13), Eqs. (10) and (11) become given by:

$$D_0 A_{0,\mathrm{x/y}} = 2iT_{0,\mathrm{x/y}}\left(c^2 k_0\frac{\partial A_{0,\mathrm{x/y}}}{\partial z} + \omega_0\frac{\partial A_{0,\mathrm{x/y}}}{\partial t}\right), \tag{14}$$

$$D_1 A_{1,\mathrm{x/y}} = 2iT_{1,\mathrm{x/y}}\left(c^2 k_1\frac{\partial A_{1,\mathrm{x/y}}}{\partial z} - \omega_1\frac{\partial A_{1,\mathrm{x/y}}}{\partial t}\right). \tag{15}$$

Using Eqs. (14) and (15), we can then recast Eqs. (1-2) as:

$$T_{0,\mathrm{x/y}}\left(c^2 k_0\frac{\partial A_{0,\mathrm{x/y}}}{\partial z} + \omega_0\frac{\partial A_{0,\mathrm{x/y}}}{\partial t}\right) = -\frac{i\omega_\mathrm{p}^2\delta\hat{n}T_{\delta n}}{2}A_{1,\mathrm{x/y}}T_{1,\mathrm{x/y}}, \tag{16}$$

$$T_{1,\mathrm{x/y}}\left(c^2k_1\frac{\partial A_{1,\mathrm{x/y}}}{\partial z}-\omega_1\frac{\partial A_{1,\mathrm{x/y}}}{\partial t}\right)=\frac{i\omega_{\mathrm{p}}^2\delta\hat{n}^*T^*_{\delta n}}{2}A_{0,\mathrm{x/y}}T_{0,\mathrm{x/y}}. \tag{17}$$

We note that Eqs. (18) and (19) generalise the well known plane wave stimulated Raman backscattering equations for arbitrary transverse laser profiles. Thus, all selection rules for the orbital angular momentum, including the creation of new modes can be derived using Eq. (3) together with Eqs. (18-19) by ensuring that all phase factors cancel.

In its most simple configuration considering lasers with identical transverse profiles in every polarisation direction, it is possible to readily recover the usual plane wave solutions. Cancellation of all phase factors leads to:

$$\left(c^2k_0\frac{\partial A_{0,\mathrm{x/y}}}{\partial z}+\omega_0\frac{\partial A_{0,\mathrm{x/y}}}{\partial t}\right)=-\frac{i\omega_{\mathrm{p}}^2\delta\hat{n}}{2}A_{1,\mathrm{x/y}}, \tag{18}$$

$$\left(c^2k_1\frac{\partial A_{1,\mathrm{x/y}}}{\partial z}-\omega_1\frac{\partial A_{1,\mathrm{x/y}}}{\partial t}\right)=\frac{i\omega_{\mathrm{p}}^2\delta\hat{n}^*}{2}A_{0,\mathrm{x/y}}. \tag{19}$$

For Laguerre Gaussian lasers with orbital angular momentum, cancelling phase factors readily implies the conservation of orbital angular momentum, $\ell_0=\ell_1+\ell_{\mathrm{p}}$, the conservation of energy, $\omega_0=\omega_1+\omega_{\mathrm{p}}$, and the conservation of linear momentum, $k_0=k_1+k_{\mathrm{p}}$.

It is possible to further simplify Eqs. (14) and (15) assuming that the spot-size does not change during propagation (a valid approximation to interpret our simulations since the Rayleigh length is much larger than the interaction length). In this case $\partial_z TA = T\partial_z A + A\partial_z T \simeq T\partial_z A$ because $A\partial_z T \ll T\partial_z A$. Thus, Eqs. (3), (14) and (15) may also take the more familiar form of the plane wave equations for Raman amplification [3, 5]:

$$\left(c^2k_0\frac{\partial}{\partial z}+\omega_0\frac{\partial}{\partial t}\right)\mathbf{A}_0\approx-\frac{i\omega_{\mathrm{p}}^2\delta n}{2}\mathbf{A}_1 \tag{20}$$

$$\left(c^2k_1\frac{\partial}{\partial z}+\omega_1\frac{\partial}{\partial t}\right)\mathbf{A}_1\approx\frac{i\omega_{\mathrm{p}}^2\delta n^*}{2}\mathbf{A}_0 \tag{21}$$

$$2i\omega_{\mathrm{p}}\frac{\partial\delta n}{\partial t}=\frac{e^2k_{\mathrm{p}}^2}{2m_e^2}\left(\mathbf{A}_0\cdot\mathbf{A}_1^*\right), \tag{22}$$

Hence the 1D spatial-temporal amplification process is independent of the transverse laser profiles, under the condition that the above selection rule for angular momentum conservation is obeyed, in addition to the usual selection rules for conservation of energy and linear momentum.

In order to derive scalings for the growth rate of the instability, we assume a long laser such that $k_{0,1}\partial\mathbf{A}_{(0,1)}/\partial z \ll \omega_{0,1}\partial\mathbf{A}_{(0,1)}/\partial t$, in order to explore the temporal problem (note

that this same approximation could be done directly to Eqs. (14) and (15) yielding the same result, without the need of assuming that $\mathbf{A}_\perp$ was a function of z only). In this case, stimulated Raman scattering equations become:

$$\omega_0 \frac{\mathrm{d}\mathbf{A}_0}{\mathrm{d}t} = -\frac{i\omega_{\mathrm{p}}^2 \delta n \mathbf{A}_1}{2}, \tag{23}$$

$$\omega_1 \frac{\mathrm{d}\mathbf{A}_1}{\mathrm{d}t} = \frac{i\omega_{\mathrm{p}}^2 \delta n^* \mathbf{A}_0}{2}, \tag{24}$$

$$\omega_{\mathrm{p}} \frac{\mathrm{d}\delta n}{\mathrm{d}t} = -\frac{ie^2 k_{\mathrm{p}}^2}{4m_e^2} \left(\mathbf{A}_1^* \cdot \mathbf{A}_0\right). \tag{25}$$

The selection rules for the orbital angular momentum, which ensure conservation of angular momentum described in the main Manuscript, also follow directly from Eqns. (23)-(25). These rules will be derived and studied in more detail below. We note that Eqs. (23)-(25) are valid for arbitrary transverse pump and seed envelope profiles.

We can derive Raman backscattering growth rates assuming that the amplitude of the pump laser remains constant during its interaction with the plasma wave and the seed laser. The latter assumption also implies that the intensity of the seed pulse is much smaller than the intensity of the pump laser. Thus, combining Eq. (24) with the time derivative of Eq. (25) and neglecting $\partial_t \mathbf{A}_0(t)$, we find:

$$\frac{\partial^2 \delta n^*}{\partial t^2} = i\frac{e^2 k_{\mathrm{p}}^2}{4\omega_{\mathrm{p}} m_e^2} \left(\mathbf{A}_0^* \cdot \frac{\partial \mathbf{A}_1}{\partial t}\right). \tag{26}$$

Equation (26) can be further simplified by substituting Eq. (24) into Eq. (26) giving:

$$\frac{\partial^2 \delta n^*}{\partial t^2} = \frac{e^2 k_{\mathrm{p}}^2 \omega_{\mathrm{p}}^2}{8\omega_{\mathrm{p}}\omega_1 m_e^2} |\mathbf{A}_0|^2 \delta n^*. \tag{27}$$

Equation (27), which can be solved exactly, determines the plasma density perturbation associated with the interaction between pump and seed. By assuming that $\delta n^*(t=0) = 0$, and that $\partial_t \delta n^*(t=0) = ie^2 k_{\mathrm{p}}^2/(4\omega_{\mathrm{p}} m_e^2)\left[\mathbf{A}_0^* \cdot \mathbf{A}_1(t=0)\right]$ (c.f. Eq. (25)), leads to:

$$\delta n^* = i\frac{e^2 k_{\mathrm{p}}^2}{4\omega_{\mathrm{p}} m_e^2}\left[\mathbf{A}_0^* \cdot \mathbf{A}_1(t=0)\right] \frac{\sinh(\Gamma t)}{\Gamma}, \tag{28}$$

$$\Gamma^2 = \frac{e^2 k_{\mathrm{p}}^2 \omega_{\mathrm{p}}^2}{8\omega_{\mathrm{p}}\omega_1 m_e^2} |\mathbf{A}_0|^2 \tag{29}$$

where Γ is the growth rate of the instability.

The temporal evolution of the seed is found by combining Eq. (28) with Eq. (24):

$$\mathbf{A}_1(t) = \left(\mathbf{A}_1(t=0) \cdot \frac{\mathbf{A}_0^*}{|\mathbf{A}_0|}\right) \frac{\mathbf{A}_0}{|\mathbf{A}_0|} \cosh(\Gamma t) + C, \tag{30}$$

where C is a constant specified by the initial seed laser vector potential profile. Equation (30) describes the temporal growth of the Raman backscattering amplified seed pulse assuming a non-evolving pump laser with an arbitrary transverse envelope.

The derivation of Eq. (30) is valid for arbitrary transverse electromagnetic field profiles of both pump and seed beams and for arbitrary pump and seed polarisations, as long as both pump and seed obey the paraxial approximation. This is a valid assumption because both Laguerre-Gaussian modes with orbital angular momentum and transverse electro-magnetic (TEM) modes are solutions of Maxwell's equations under the paraxial equation, valid as long as the transverse size of the laser is much larger than the laser wavelength at the focal plane, or, more generally, valid in the limit of small angles from the axis. Equations (29) and (30) then show that the growth rates are fully determined by the intensity profile of the pump. The initial seed for stimulated Raman scattering, which determines the level of amplification as a function of the interaction length, will depend on the initial overlap between pump and probe according to Eq. (30).

The main assumption of Eqs. (26)-(30) is that the pump energy does not change during the interaction. Although this assumption is strictly valid at sufficiently early times when compared with the growth rates, the model accurately predicts all selection rules, together with the creation and amplification of new OAM modes. We have confirmed all these predictions using 3D PIC simulations. Therefore inclusion of pump depletion effects will not affect the phenomenology for the OAM beam amplification, although they may change the growth rates according to Ref. [6].

SUPPLEMENTARY NOTE 2 - AMPLIFICATION OF AN EXISTING OAM MODE

In this section, we study the Raman amplification of laser pulses with orbital angular momentum, and derive selection rules for the OAM of the pump and seed pulses and the plasma wave. In this and the subsequent sections, we assume that each laser with OAM ℓ_{laser} can be described as $\mathbf{a}_{\mathrm{laser}} \sim \exp(i\ell_{\mathrm{laser}}\phi)$ and each plasma wave OAM mode can be described by $\delta n \sim \exp(i\ell_{\mathrm{p}}\phi)$.

We start by studying the case leading to the amplification of an existing OAM seed. We then consider a seed laser with OAM component given by $\mathbf{a}_1 \sim \exp\left(i\ell_{1\mathrm{x}}\phi\right)\mathbf{e}_x$, and a pump with $\mathbf{a}_0 \sim \exp\left(i\ell_{0\mathrm{x}}\phi\right)\mathbf{e}_x$ where $\mathbf{e}_x$ is the unit vector in the x direction, indicating the direction

of polarisation. Direct substituion in Eq. (25) shows that the pump and the seed create a OAM plasma wave perturbation with $\delta n \sim \exp(i\ell_{\rm p}\phi)$. Thus, the OAM of the plasma wave is $\ell_{\rm p} = \ell_{\rm 0x} - \ell_{\rm 1x}$, ensuring angular momentum conservation. The same selection rule has also been derived above in Eqs. (23) and (24), proving that these equations are mutually consistent. Thus, a pump with a single, but arbitrary OAM mode, or even without any OAM at all, can be used to amplify a seed pulse with a single and also arbitrary OAM mode, because the plasma wave will carry all excess angular momentum. This is schematically shown in Fig. 1, which illustrates several examples of stimulated Raman amplification for various combinations of pump/seed OAM modes. Figure 1(a) then shows the matching conditions for a Gaussian pump and seed, where only the longitudinal wavenumber matching conditions need to be satisfied. Figure 1b shows the matching conditions for the amplification of an OAM seed by a Gaussian pump. Amplification also occurs in this case because the plasma wave carries excess angular momentum from the seed, ensuring the conservation of angular momentum. This setup is relevant because it shows that the amplification of an OAM seed beam does not necessarily requires a pump with OAM. Figures 1(c)-(d) show additional configurations leading to the amplification of an OAM seed.

A similar calculation can be performed for the case of circular polarisation, leading to the same selection rules.

SUPPLEMENTARY NOTE 3 - GENERATION OF NEW OAM MODES

In order to explore the generation and amplification of new OAM modes in the seed, we consider a pump with $\mathbf{a}_0 \sim \exp(i\ell_{\rm 0x}\phi)\mathbf{e}_x + \exp(i\ell_{\rm 0y}\phi)\mathbf{e}_y$, and a seed with $\mathbf{a}_1 \sim \exp(i\ell_{\rm 1x}\phi)\mathbf{e}_x$. Hence, the pump has an OAM mode linearly polarised in each transverse direction, and the seed contains a single OAM mode linearly polarised in the x direction. According to Eq. (25), the seed component in x interacts with the pump component also polarised in x, generating a plasma wave with $\ell_{\rm p} = \ell_{\rm 0x} - \ell_{\rm 1x}$. This daughter plasma wave can interact with the pump component polarised in the y direction, leading to the generation of a new seed OAM mode polarised in the y direction with $\ell_{\rm 1y} = \ell_{\rm 0y} - \ell_p = \ell_{\rm 0y} - \ell_{\rm 0x} + \ell_{\rm 1x}$, in agreement with Eq. (30).

Figure 2 illustrates this step where the matching conditions are satisfied for the x direction from the start of the interaction for a particular case where the pump OAM in x is $\ell_{\rm 0x} = 0$

and in y is $\ell_{0\mathrm{y}} = 1$, and where the initial seed is polarised in x with $\ell_{1\mathrm{x}} = 1$. In Fig. 2(a), the x pump component interacts with the existing OAM seed component in x to produce a plasma wave with OAM given by $\ell_{\mathrm{p}} = \ell_{0\mathrm{x}} - \ell_{1\mathrm{x}} = -1$ (green dashed line). Figure 2(b) illustrates this second step where the matching conditions are satisfied for the y direction once a new seed is created in that direction. In Fig. 2(b), the plasma wave then interacts with the pump polarised in y and lead to a new seed polarised in y with $\ell_{1\mathrm{y}} = \ell_{0\mathrm{y}} - \ell_p = 2$ (orange dashed line) In addition, the existing seed mode linearly polarised in x $[\exp(i\ell_{1\mathrm{x}}\phi)]$ will continue to be amplified. Figure 2 illustrates these steps leading to the generation of a new linearly polarised OAM seed. It is important to note that the new $\ell_{1\mathrm{y}}$ mode in the seed cannot interact with the $\ell_{0\mathrm{x}}$ mode in the pump since these modes have orthogonal polarisation.

An identical setup can also be used to generate and amplify a new mode with circular polarisation. This is possible because right-handed and left-handed circularly polarised modes do not interact, just as linearly polarised modes in two orthogonal directions do not interact. We now consider an initial pump with $\mathbf{a}_0 \sim \exp(i\ell_{0+}\phi)\,(\mathbf{e}_x + i\mathbf{e}_y) + \exp(i\ell_{0-}\phi)\,(\mathbf{e}_x - i\mathbf{e}_y)$ and an initial seed with $\mathbf{a}_1 \sim \exp(i\ell_{1+}\phi)\,(\mathbf{e}_x + i\mathbf{e}_y)$. Hence, the pump has a single OAM mode ℓ_{0+} circularly polarised in $\mathbf{e}_+$ and a single OAM mode ℓ_{0-} circularly polarised in $\mathbf{e}_-$. The seed, with an initial single OAM mode ℓ_{1+}, is circularly polarised in the anti-clock wise direction. According to Eq. (30), the anti-clock wise seed and pump generate a plasma wave with $\ell_{\mathrm{p}} = \ell_{0+} - \ell_{1+}$. The plasma wave then interacts with the pump circularly polarised in the clockwise direction and produces a new seed mode, also circularly polarised in the clockwise direction with $\ell_{1-} = \ell_{0-} - \ell_p = \ell_{0-} - \ell_{0+} + \ell_{1+}$. Direct substitution of pump and seed expressions in Eq. (30) then yields $\mathbf{a}_1 \sim \exp(i\ell_{1+}\phi)\,(\mathbf{e}_x + i\mathbf{e}_y) + \exp\left[i\left(-\ell_{0\mathrm{x}} + \ell_{1\mathrm{x}} + \ell_{0\mathrm{y}}\right)\phi\right](\mathbf{e}_x - i\mathbf{e}_y)$, consistent with the conservation of orbital angular momentum. As a result, and in addition to the amplification of the existing circularly polarised mode, a new circularly polarised OAM seed can also be produced and amplified with a handedness opposite to the initial seed.

SUPPLEMENTARY NOTE 4 - CONVERSION FROM TEM TO OAM MODES

Figure 3 shows the generation of OAM from initial TEM modes, where we consider a pump given by $\mathbf{a}_0 \sim \mathrm{TEM}_{00}\,(\mathbf{e}_x + \mathbf{e}_y)$, and an initial seed given by $\mathbf{a}_1 \sim \mathrm{TEM}_{10}\mathbf{e}_x + i\mathrm{TEM}_{01}\mathbf{e}_y$, i.e. the y seed component is $\pi/2$ out of phase with respect to the seed x com-

ponent. Initial pump modes are shown in Figs. 3(a) and (c). The TEM_{00} pump polarised in x then interacts with the TEM_{10} seed also polarised in x to generate a TEM plasma wave with TEM_{10} (plasma wave represented by the green dashed lines in Fig. 3(a)). This daughter plasma wave then interacts with the TEM_{00} pump polarised in y yielding a new TEM_{10} seed mode polarised in y (new seed mode in y represented by the orange dashed lines in Fig. 3(b)). Simultaneously, the TEM_{00} pump polarised in y interacts with the $i\mathrm{TEM}_{01}$ seed also polarised in y to generate a plasma wave with $i\mathrm{TEM}_{01}$. (new plasma wave mode represented in dashed green in Fig. 3c). This daughter plasma wave then interacts with the TEM_{00} pump polarised in x and generates a new $i\mathrm{TEM}_{01}$ seed polarised in x (new seed in x represented by orange dashed lines in Fig. 3d). As a result, a new seed is created and amplified with $\mathbf{a}_1 \sim (\mathrm{TEM}_{10} + i\mathrm{TEM}_{01})(\mathbf{e}_x + \mathbf{e}_y)$. According to Ref. [7], this corresponds to new OAM modes with $\ell_{1\mathrm{x}} = \ell_{1\mathrm{y}} = 1$. Equivalently, substituting pump and seed expressions into Eq. (30) leads to $\mathbf{a}_2 \sim \mathrm{TEM}_{00}\,(\mathrm{TEM}_{10} + i\mathrm{TEM}_{01})\,(\mathbf{e}_x + \mathbf{e}_y)$, where $\mathrm{TEM}_{00} \sim 1$ in this context.

An alternative way to view this scheme is to define new ortogonal unit vectors given by $\mathbf{e}_{1,2} = (\mathbf{e}_x \pm \mathbf{e}_y)/\sqrt{2}$ and write the seed pulse as $\mathbf{a}_1 \sim (\mathrm{TEM}_{10} + i\mathrm{TEM}_{01})\mathbf{e}_1 + (\mathrm{TEM}_{10} - i\mathrm{TEM}_{01})\mathbf{e}_2$. While this pulse has no overall OAM, the $\mathbf{e}_1$ and $\mathbf{e}_2$ components have OAM of $\ell = 1$ and $\ell = -1$ respectively. Since the pump pulse is given by $\mathbf{a}_0 \sim \mathrm{TEM}_{00}\mathbf{e}_1$, it will amplify the $\ell = 1$ mode (same polarisation) while leaving the $\ell = -1$ mode untouched (orthogonal polarisation). The amplified seed pulse will then end up with overal OAM of level $\ell = 1$, while it had no OAM initially. This example also illustrates that it is possible to selectively amplify OAM modes in the seed pulse based on their polarisation relative to the polarisation of the pump pulse.

SUPPLEMENTARY NOTE 5 - PLASMAS AND KERR MEDIA

In stimulated Raman scattering in plasmas the electromagnetic pump wave decays into a scattered electromagnetic wave with the Langmuir wave providing the coupling between the other two electromagnetic waves. In other nonlinear optical media, the role of the Langmuir wave would be replaced by molecular vibrations, for example. Equations for three wave

mixing processes in nonlinear anisotropic optical media with Kerr nonlinearity are [8]:

$$\frac{\mathrm{d}A_i^{(3)}}{\mathrm{d}z} = i\frac{k_3}{n^2}\mathrm{d}_{ijk}A_j^{(1)}A_k^{(2)} \tag{31}$$

$$\frac{\mathrm{d}A_i^{(1)}}{\mathrm{d}z} = i\frac{k_1}{n^2}\mathrm{d}_{ijk}A_j^{(3)}A_k^{(2)*} \tag{32}$$

$$\frac{\mathrm{d}A_i^{(2)}}{\mathrm{d}z} = i\frac{k_2}{n^2}\mathrm{d}_{ijk}A_j^{(3)}A_k^{(1)*}, \tag{33}$$

where $A_i^{(m)}$ is the electromagnetic field envelope component in the $x(i = 1)$, $y(i = 2)$, and $z(i = 3)$ direction. The superscript $m = 1, 2, 3$ defines each of the three waves that participate in the process. In addition, z is the propagation direction, (k_1, k_2, k_3) are the laser wavenumbers of each laser, $n = \sqrt{1 + \chi_0}$ with χ_0 being the linear susceptibility, and the d_{ikj} are the susceptibility coefficients. Eq. (31)-(33) assumes frequency and wavenumber matching conditions given by $\omega_3 = \omega_1 + \omega_2$ and $k_3 = k_1 + k_2$.

We now assume that $A_i^{(2)}$ is now polarised in x (i.e. $A_1^{(2)} \neq 0$, $A_2^{(2)} = A_3^{(2)} = 0$) with the role of being an idler beam that will cary the excess angular momentum between the pump and the seed, analogous to the electron plasma wave in this respect. In this case Eq. (31)-(33) is formally identical to Eqs. (23)-(25) as long as $\mathrm{d}_{111} = \mathrm{d}_{221} = \mathrm{d}_{122} = d \neq 0$, with $d_{ijk} = 0$ for any other combination of the triad (ijk) with $i \neq 3$, since z $(i = 3)$ is the propagation direction. In these conditions, Eqs. (31)-(33) become:

$$\frac{\mathrm{d}\mathbf{A}^{(3)}}{\mathrm{d}z} = id\frac{k_3}{n^2}A_1^{(2)}\mathbf{A}^{(1)} \tag{34}$$

$$\frac{\mathrm{d}\mathbf{A}^{(1)}}{\mathrm{d}z} = id\frac{k_1}{n^2}A_1^{(2)*}\mathbf{A}^{(3)} \tag{35}$$

$$\frac{\mathrm{d}A^{(2)}}{\mathrm{d}z} = id\frac{k_2}{n^2}\mathbf{A}^{(3)} \cdot \mathbf{A}^{(1)*}. \tag{36}$$

Equations (34)-(36) are identical to the stimulated Raman backscattering in a plasma given by Eqs. (23)-(25). If $\mathrm{d}_{111} \neq \mathrm{d}_{221} \neq \mathrm{d}_{122}$, the coupling between the three waves will differ from the plasma case. If other non-zero components of d_{ijk} appear, then the coupling between each wave in the different polarisation directions will also differ from the plasma case. Still the ideas and principles established in our work will still hold. The plasma, under the approximations we have performed and for which Eq. (23)-(25) are valid, is therefore a special case of a non-linear optical medium.

SUPPLEMENTARY REFERENCES

[1] D.W. Forslund, J.M. Kindel, E.L. Lindman, Phys. Fluis **18**, 1002 (1975).

[2] G. Shvets *et al*, Superradiant Amplification of an Ultrashort Laser Pulse in a Plasma by a Counterpropagating Pump, Phys. Rev. Lett. **81**, 4879 (1998).

[3] V.M. Malkin *et al*, Fast Compression of Laser Beams to Highly Overcritical Powers, Phys. Rev. Lett. **82**, 4448 (1999).

[4] J.T. Mendonça, B. Thidé, H. Then, Stimulated Raman and Brillouin Backscattering of Collimated Beams Carrying Orbital Angular Momentum, Phys. Rev. Lett. **102**, 185005 (2009).

[5] V.M. Malkin *et al*, Ultra-powerful compact amplifiers for short laser pulses, Phys. Plasmas **7**, 2232 (2000).

[6] R.M.G.M. Trines *et al*, Production of Picosecond, Kilojoule, and Petawatt Laser Pulses via Raman Amplification of Nanosecond Pulses, Phys. Rev. Lett. **107**, 105002 (2011).

[7] L. Allen, W. Beijersbergen, R.J.C. Spreeuw, J.P. Woerdman, Orbital angular momentum of light and the transformation of Laguerre-Gaussian laser modes, Phys. Rev. A **45**, 8185 (1992).

[8] Applications of Classical Physics, Roger D. Blandford and Kip S. Thorne, http://www.pmaweb.caltech.edu/Courses/ph136/yr2012/1210.1.K.pdf (2012).
